# Supplementary material for: Experimental demonstration of adversarial examples in learning topological phases
Source: Nat Commun. 2022 Aug 25;13:4993. doi: 10.1038/s41467-022-32611-7 (PMC9411630; doi:10.1038/s41467-022-32611-7)
Supplement: Supplementary file 1 — Supplementary Information [file 41467_2022_32611_MOESM1_ESM.pdf]

Supplementary Information: Experimental demonstration of adversarial examples in learning topological phases

Huili Zhang,[1, *] Si Jiang,[1, *] Xin Wang,[1, *] Wengang Zhang,[1] Xianzhi Huang,[1, 2] Xiaolong Ouyang,[1] Yefei Yu,[1] Yanqing Liu,[1] Dong-Ling Deng,[1, 3, †] and L.-M. Duan[1, ‡]

[1] *Center for Quantum Information, IIIS, Tsinghua University, Beijing 100084, P. R. China*
[2] *School of JiaYang, Zhejiang Shuren University,Hangzhou 310015, P. R. China*
[3] *Shanghai Qi Zhi Institute, 41th Floor, AI Tower, No. 701 Yunjin Road, Xuhui District, Shanghai 200232, China*
(Dated: July 27, 2022)

SUPPLEMENTARY NOTE 1: EXPERIMENTAL SETUP

This experiment is implemented on a home-built confocal microscope at room temperature. The diamond sample (type IIa, Element 6) is grown via chemical vapor deposition and cut along ⟨100⟩ orientation, with the natural abundance of 1.1% ^{13}C. The 532 nm diode laser (Coherent Compass 315M) passes through an acoustic optical modulator (AOM, Isomet 1250C-848) setting in a double-pass configuration. This configuration can increase the on-off ratio to 10000:1 and constrain the leakage of the laser. Then the laser is reflected by a diachronic mirror (DM) into an oil-immersed objective lens (NA=1.49, Olympus), and the focused spot size is about 300×300 nm^2 on the diamond. The lens is mounted on a three-axis closed-loop piezo (Physik Instrumente, E-725) with a scanning range of $100\times100\times100$ μm^3. The photons in the wavelength ranging from 637 to 800 nm pass through the same objective lens and DM, then are collected by a single-mode fiber and detected by a single photon counting module (SPCM, Excelitas, SPCM-AQRH-14-FC). A permanent magnetic provides the static magnetic field of 472 Gauss, by observing the emitted photon numbers, we can align the magnetic field parallel to the NV symmetry axis. The magnitic field removes the degeneracy between $|m_s = \pm 1\rangle$ states and polarizes the nuclear spin with the polarization rate typically exceeding 95% [1].

The microwave (MW) is generated by an analog signal generator (Agilent, N5181B) and modulated by an IQ mixer (Marki IQ 1545LMP) with two orthogonal 100 MHz carrier signals, which are generated from the analog output of an arbitrary waveform generator (AWG,Tektronic 5014C, sample rate 1 GHz). The combined signal then is amplified by a high power amplifier (Mini Circuits, ZHL-16w-43-S+) and delivered into a gold coplanar waveguide close to the NV center. The amplitude of the combined signal is in the linear range of the amplifier to ensure that the Rabi frequency is proportional to the amplitude of the combined signal.

In our experiment, a single sequence starts with 3 μs laser excitation for the electron spin polarization and ends with 3 μs laser pulse for the spin state detection. The signal photons are collected for 200 ns right after the detection laser rises and reference photons are collected for 200 ns after 2 μs. The sequences are programmed and loaded to the AWG. To enhance collection efficiency, a solid immersion lens with 6.74 μm diameter is fabricated by a focused ion beam (FEI company, Helios nanolab 660). The photon number is about 260 kcps under 0.25 mW laser excitation, increasing the signal-noise ratio to about $100:1$. The sequence is repeated 7.5×10^5 times, collecting about 3.9×10^4 photons.

SUPPLEMENTARY NOTE 2: ADIABATIC PASSAGE

Consider the electron subspace of $|0\rangle$ and $|-1\rangle$ states, in a rotating frame, the effective Hamiltonian with variable time t is

$$H(t) = \Omega(t)(S_x \cos\varphi - S_y \sin\varphi) + \Delta\omega(t) S_z, \quad (1)$$

where $\Omega(t)$ is the MW amplitude, φ is the MW phase, and $\Delta\omega(t) = \omega_0 - \omega_{\mathrm{MW}}$, with ω_0 and ω_{MW} being the resonant frequency and MW frequency, respectively; $S_{x,y,z}$ are given as the following (setting $\hbar = 1$):

$$S_x = \frac{1}{2}\begin{pmatrix} 0 & 1 \\ 1 & 0 \end{pmatrix}, S_y = \frac{1}{2}\begin{pmatrix} 0 & -i \\ i & 0 \end{pmatrix}, S_z = \begin{pmatrix} 0 & 0 \\ 0 & -1 \end{pmatrix}. \quad (2)$$

We apply the adiabatic passage process described as

$$\Omega(t) = \begin{cases} 2\Omega_{\max} t/T, & t \le T/2, \\ 2\Omega_{\max}(1 - t/T) & t > T/2, \end{cases} \quad (3)$$

$$\Delta\omega(t) = \Delta\omega_{\max} - 2\Delta\omega_{\max}\ t/T. \quad (4)$$

The frequency and amplitude are shown in Supplementary Fig. 1.

Comparing with the Hamiltonian in Supplementary Eq. (1), we terminate the adiabatic passage process at time t_c to satisfy $\Delta\omega(t_c)/\Omega(t_c) = u_z/\sqrt{u_x^2 + u_y^2}$. Phase

---
* These authors contributed equally to this work.
† dldeng@tsinghua.edu.cn
‡ lmduan@tsinghua.edu.cn

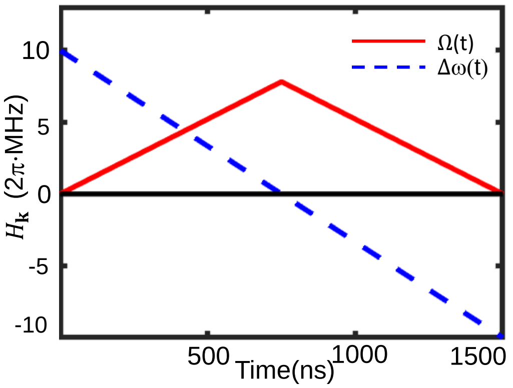

Supplementary Figure 1. **The MW frequency and amplitude in the adiabatic passage.**

$\varphi = -\arctan(u_y/u_x)$ is kept constant during the adiabatic passage process. To satisfy the adiabatic condition [2]:

$$Q \equiv \frac{2(\Delta\omega(t)^2 + |\Omega_2(t)|^2)^{3/2}}{||\dot{\Omega}(t)|\Delta\omega(t) - |\Omega(t)|\dot{\Delta\omega}(t)|} \gg 1, \tag{5}$$

in our experiment, we use $\Omega_{\max} = 2\pi \times 7.81$ MHz and $\Delta\omega_{\max} = 2\pi \times 10$ MHz. During the adiabatic passage process, we keep $Q > 25$ in our experiment.

SUPPLEMENTARY NOTE 3:. QUANTUM STATE TOMOGRAPHY

We perform a full quantum state tomography to measure the eigenstates of the topological Hamiltonian at each momentum **k**. The photon numbers in the basis {Z} is directly measured and in bases {+X, +Y, −X}, a $\pi/2$ pulse with phase $\{0, -\pi/2, \pi\}$ will be inserted before the detection. In a single-qubit system, to ensure the non-negative definite, Hermitian, and trace-one properties of a density matrix, the density matrix $\hat{\rho}$ can be written as $\hat{\rho}_t = T^\dagger(t)T(t)/\mathrm{Tr}\{T^\dagger(t)T(t)\}$, where T reads:

$$T(t) = \begin{pmatrix} t_1 & 0 \\ t_2 + it_3 & t_4 \end{pmatrix}. \tag{6}$$

The measurement consists of a set of four coincidence photon numbers. In our experiment the expected photon number for the ν-th measurement is

$$\bar{n}_\nu(t_1, t_2, t_3, t_4) = N_0 p_{0,\nu} + N_{-1}(1 - p_{0,\nu}), \tag{7}$$

where N_0 and N_{-1} are the photon numbers of states $|0\rangle$ and $|-1\rangle$ measured by fast Rabi oscillation, and p_0 denotes the probability for the spin at $|0\rangle$. Assuming the noise on this coincidence measurements has a Gaussian probability distribution, the probability $P(n_1, n_2, n_3, n_4)$ of photon numbers $\{n_1, n_2, n_3, n_4\}$ produced by the density matrix $\hat{\rho}_p(t_1, t_2, t_3, t_4)$ reads:

$$P(n_1, n_2, n_3, n_4) = \prod_{\nu=1}^{4} \exp\left[-\frac{(n_\nu - \bar{n}_\nu)^2}{2\sigma_\nu^2} \right], \tag{8}$$

where σ_ν is the standard deviation of photon numbers for the ν-th measurement (approximated to be $\sqrt{\bar{n}_\nu}$) and n_ν is photon numbers measured in different tomography bases. The optimization problem reduces to finding the minimum of the following function [3]:

$$\mathcal{L}(t_1, t_2, t_3, t_4) = \sum_{\nu=1}^{4} \frac{[\bar{n}_\nu(t_1, t_2, t_3, t_4) - n_\nu]^2}{2\bar{n}_\nu(t_1, t_2, t_3, t_4)}. \tag{9}$$

Error bars are calculated through Monte Carlo simulations by assuming a Poissonian distribution of the photon numbers. Supplementary Fig. 2 and Supplementary Fig. 3**a** show the average fidelity for different Hopf index for legitimate samples and adversarial examples, respectively. For legitimate samples, the average fidelity for $h = 3.2$, $h = 2$ and $h = 0.5$ are 99.77(41) %, 99.78(41) %, and 99.77(45) %, respectively. Similarly, for adversarial examples, the average fidelity for $h = 3.2$, $h = 2$ and $h = 0.5$ are 99.64(43) %, 99.65(46) %, and 99.48(46) %, respectively. We also show the average fidelity for each k_z value with $h = 0.5$ and $h = 3.2$ between the numerically generated and experimentally implemented states in Supplementary Fig. 3**b - c**.

SUPPLEMENTARY NOTE 4:. DISCRETIZATION SCHEME TO MEASURE HOPF INDEX

We use the discretization scheme introduced in [4, 5] and applied in [6] to measure the Hopf index directly from experimental data. The Hopf index can be written as:

$$\chi = -\int_{\mathrm{BZ}} \boldsymbol{F} \cdot \boldsymbol{A} d^3\mathbf{k}, \tag{10}$$

where $\boldsymbol{F}$ is the Berry curvature with $F_\mu = \frac{i}{2\pi}\epsilon_{\mu\nu\tau}(\partial_{k_\nu}\langle\psi_{\mathbf{k}}|)(\partial_{k_\tau}|\psi_{\mathbf{k}}\rangle)$ and $\boldsymbol{A}$ is the associated Berry connection satisfying $\nabla \times \boldsymbol{A} = \boldsymbol{F}$. To avoid the arbitrary phase problem, we can use a discretized version of the Berry curvature [4, 5]:

$$F_\mu(\mathbf{k}_{\boldsymbol{J}}) = \frac{i}{2\pi}\epsilon_{\mu\nu\tau} \ln U_\nu(\mathbf{k}_{\boldsymbol{J}}) \ln U_\tau(\mathbf{k}_{\boldsymbol{J}+\hat{\nu}}). \tag{11}$$

The $U(1)$-link is defined as

$$U_\nu(\mathbf{k}_{\boldsymbol{J}}) = \frac{\langle\psi(\mathbf{k}_{\boldsymbol{J}})|\psi(\mathbf{k}_{\boldsymbol{J}+\hat{\nu}})\rangle}{|\langle\psi(\mathbf{k}_{\boldsymbol{J}})|\psi(\mathbf{k}_{\boldsymbol{J}+\hat{\nu}})\rangle|}, \tag{12}$$

with $\hat{\boldsymbol{\nu}} \in \{\hat{\boldsymbol{x}}, \hat{\boldsymbol{y}}, \hat{\boldsymbol{z}}\}$, which is a unit vector in the corresponding direction. This discretized version of $\boldsymbol{F}$ can be

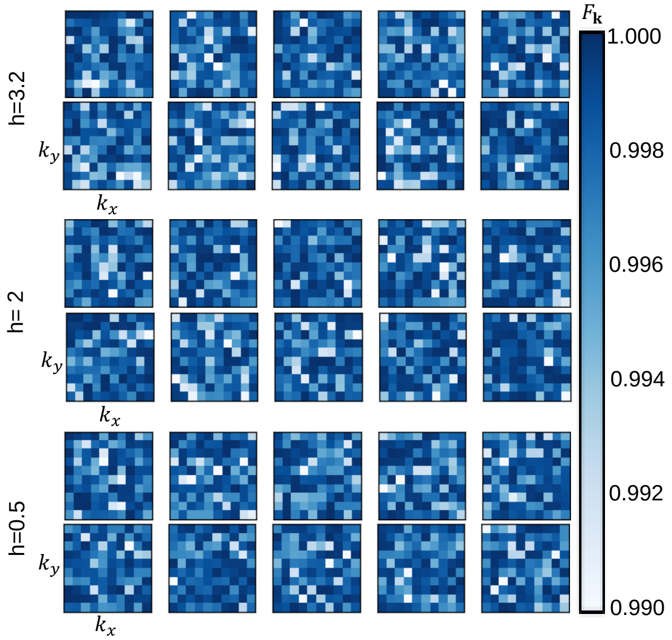

Supplementary Figure 2. **State fidelity $F_{\mathbf{k}}$ for legitimate samples.** The states are measured via the likelihood estimation at different momenta $\mathbf{k}$. Each panel consists of 10 subfigures where the momenta k_z are equally spaced from 0 to 1.8π. The horizontal and vertical axes of each subfigure denote k_x and k_y varying from 0 to 1.8π with equal spacing. The panels represent parameters $h = 3.2$ with average fidelity 99.77(41)%, h =2 with average fidelity 99.78(41)% and $h = 0.5$ with average fidelity 99.77(45)%, respectively.

calculated after performing quantum state tomography at all points $\mathbf{k}_{\boldsymbol{J}}$ on the momentum grid. We can also obtain the Berry connection $\boldsymbol{A}$ by Fourier transforming $\nabla \times \boldsymbol{A} = \boldsymbol{F}$ with the Coulomb gauge $\nabla \cdot \boldsymbol{A} = 0$. Finally, instead of doing the integral, we sum over all points on the momentum grid to obtain the Hopf index χ. It is shown in [2, 6] that for a $10 \times 10 \times 10$ grid, this method is quite robust to various perturbations and can extract the Hopf index with high accuracy.

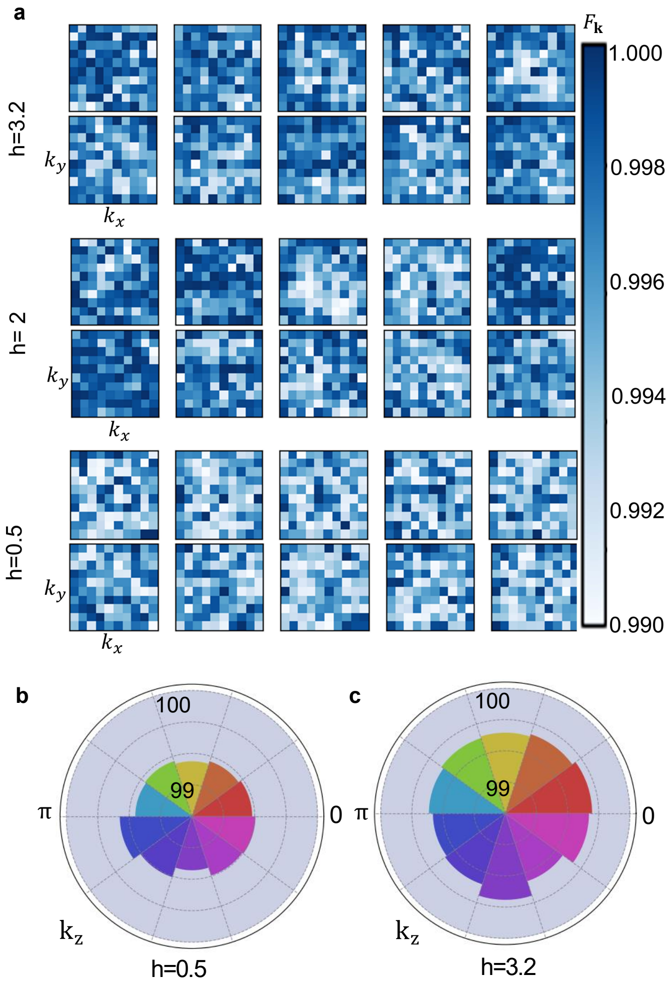

Supplementary Figure 3. **State fidelity $F_{\mathbf{k}}$ for adversarial examples. a** The states are measured via the likelihood estimation at different momenta $\mathbf{k}$. Each panel plots 10 subfigures with the momenta k_z equally spaced from 0 to 1.8π. The horizontal and vertical axes of each subfigure denote k_x and k_y varying from 0 to 1.8π with equal spacing. The panels represent parameters $h = 3.2$ with average fidelity 99.64(43)%, h =2 with average fidelity 99.65(46)% and $h = 0.5$ with average fidelity 99.48(46)%, respectively. **b** The average fidelities at different $\mathbf{k_z}$ values for $h = 0.5$. The angular direction represents different k_z and the radius direction represents the fidelity. **c** The average fidelities at different $\mathbf{k_z}$ values for and $h = 3.2$.

SUPPLEMENTARY NOTE 5: CONVOLUTIONAL NEURAL NETWORK CLASSIFIER

We use a 3D convolutional neural network (CNN) classifier to predict the Hopf index. The classifier accepts density matrices on a 10×10×10 momentum grid as input. Each density matrix is represented by three real indices (x_1, x_2, x_3) in the Bloch sphere, where

$$x_i = \mathrm{tr}(\rho\sigma_i), \quad i = 1, 2, 3, \tag{13}$$

with σ_i $(i = 1, 2, 3)$ denoting the usual Pauli matrices. With the activation function set as the Relu function, the classifier consists of an input layer with the shape 10×10×10×3, two 3D convolution layers, one max pooling layer, and one fully-connected flattening layer. The classifier ends with a softmax layer, which outputs the classification confidences $P(\chi = 0, 1, -2)$ for each topological phase. The detail of parameters used in the classifier are listed in the Supplementary Table 1.

We adapt the supervised learning approach to training our classifier learning topological phases [7–9]. With h uniformly varied from -5 to 5, we numerically generate 5000 samples with known Hopf index χ. To avoid the numerically generated data being too close to experimental

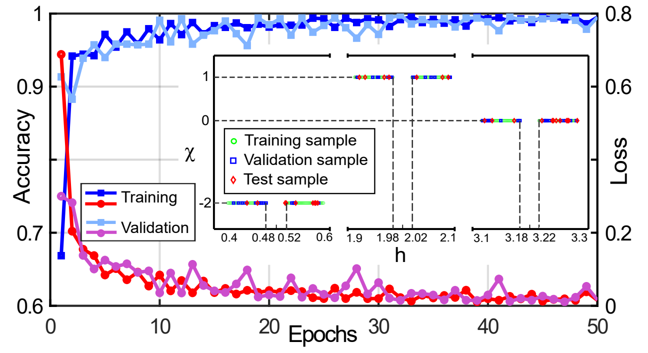

Supplementary Figure 4. **The accuracy and cross-entropy at different epochs in the training process.** The classifier is trained on numerically generated data from $h \in [-5, 5]$ except intervals $[0.48, 0.52]$, $[1.98, 2.02]$ and $[3.18, 3.22]$. There are 3471 samples in the training set, 952 samples in the validation set, and 521 samples in the test set. The inset shows the distribution of the training, validation, and test samples. The accuracy and cross-entropy loss converge after about 10 epochs.

legitimate samples with $h = 0.5, 2, 3.2$, we remove numerical samples in the intervals $[0.48, 0.52]$, $[1.98, 2.02]$, and $[3.18, 3.22]$, where the experimental legitimate samples lie. We randomly choose samples to form a training set with size 3471, a validation set with size 952, and a test set with size 521. The accuracy and cross-entropy at different epochs in the training process are shown in Supplementary Fig. 4 and the inset shows the distribution of the training, validation, and test samples. The hyper-parameters used in the training process are shown in the Supplementary Table 2.

Layer	Size/Ch.	Stride	Act.
Input	3		
Conv3D $2 \times 2 \times 2$	8	1	Relu
Conv3D $4 \times 4 \times 4$	16	1	Relu
MaxPool $2 \times 2 \times 2$		1	
Flatten			
Linear	512		Relu
Linear	3		Softmax

Supplementary Table 1. **The architecture details of the classifier.** Size represents the number of the hidden nodes used in fully-connected layers. Ch. represents the number of channels used in convolutional layers. Act. represents the activation function.

Optimizer	Epoch	Batch size	Learning rate	ρ (in RMSprop)
RMSprop	50	128	10^{-3}	0.9

Supplementary Table 2. **The hyper-parameters used for training the classifier.**

The training process ends with an accuracy of 99.2 % on the training set and 99.6 % on the validation set. On the test set, the classifier obtains an accuracy of 99.2 % and the outputs for each sample are shown in Supplementary Fig. 5. One can identify the correct phase transition points $h = -3, -1, 1, 3$ from the sharp probabilities crosses.

SUPPLEMENTARY NOTE 6: ADVERSARIAL EXAMPLES GENERATION

In the main text, we claim that our CNN classifier is vulnerable to adversarial examples, which are obtained by adding a tiny but carefully-crafted perturbation on the legitimate data. In the supervised learning case, as the legitimate samples are labeled by $(\mathbf{x}_{\text{leg}}, y_{\text{leg}})$, the task to generate adversarial examples reduces to maximize the loss function on legitimate samples:

$$\max_{\boldsymbol{\delta} \in \Delta} L(f(\boldsymbol{x}_{\text{leg}} + \boldsymbol{\delta}; \theta), y_{\text{leg}}). \tag{14}$$

In this work, we apply three typical methods in the adversarial machine learning literature to solve this problem: projected gradient descent (PGD) [10] and momentum iterative method (MIM) [11] for the continuous attack scenario; differential evolution algorithm (DEA) [12, 13] for the discrete attack scenario.

Projected gradient descent

Projected gradient descent (PGD) is developed based on an elementary method called the fast gradient sign method (FGSM) [10]. The FGSM is a one-step attack that perturbs the legitimate sample $\boldsymbol{x}_{\text{leg}}$ according to the sign of the gradient:

$$\delta_{\text{FGSM}} = \epsilon \cdot \text{sign}(\nabla_x L(f(\boldsymbol{x}_{\text{leg}}; \theta), y_{\text{leg}})). \tag{15}$$

With a large step size ϵ, the FGSM may perform poorly at the point whose gradient changes abruptly. To deal with this problem, PGD uses FGSM with a multiple-step procedure. In each step, PGD performs a projection π_C to ensure that the perturbation is restricted in a certain region [10]:

$$\boldsymbol{x}_{\text{leg}}^{(t+1)} = \pi_C(\boldsymbol{x}_{\text{leg}}^{(t)} + \frac{\epsilon}{T} \cdot \delta_{\text{FGSM}}(\boldsymbol{x}_{\text{leg}}^{(t)})), \quad t = 1, 2 \ldots, T. \tag{16}$$

In our work, we restrict the perturbation to be within the region with l_∞-norm γ, which means that for each component x_j of $\boldsymbol{x}_{\text{leg}}$, π_C projects it into $[x_{\text{leg},j} - \gamma, x_{\text{leg},j} + \gamma]$. The pseudo-code for PGD is shown in Algorithm 1.

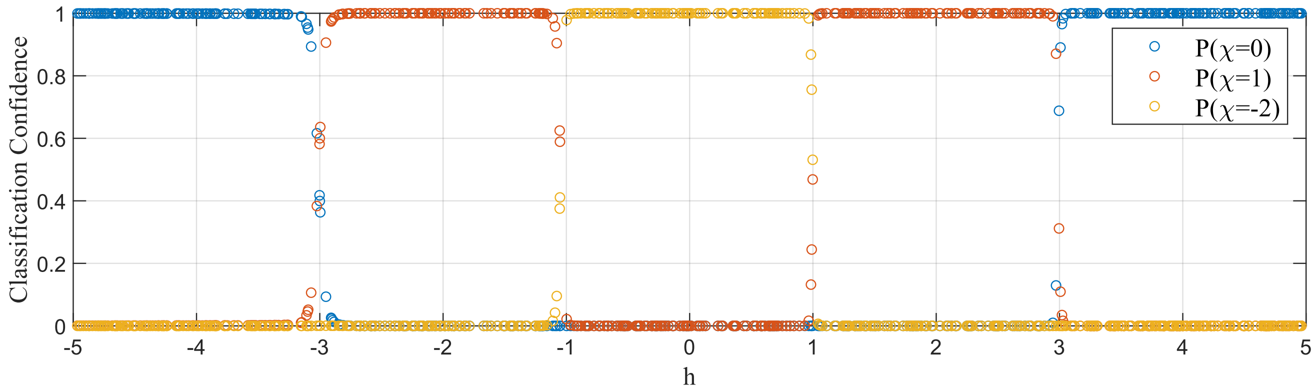

Supplementary Figure 5. **The classifier's output on the test set.** For each input as the density matrices on the $10 \times 10 \times 10$ momentum grid, the classifier outputs the probabilities for each phase that the input may belong to. The classifier exhibits near-perfect performance on the data which it has not seen before, and successfully identify the transition points by the sharp confidence clips around $|h| = 3$ and 1.

Algorithm 1 Projected Gradient Descent Method

Input The classifier $f(\bullet;\theta)$, loss function L, the legitimate density matrix $(\boldsymbol{x}_{\text{leg}}, y)$.
Input The FGSM step size ϵ, iteration number T, l_∞-norm restriction γ.
Output An adversarial example $\boldsymbol{x}^*$.

$\boldsymbol{x}^{(0)} = \boldsymbol{x}_{\text{leg}}$
$\alpha = \frac{\epsilon}{T}$
for $i = 1, \ldots, T$ **do**
  $g^{(i)} = \nabla_x L(f(\boldsymbol{x}^{(i-1)};\theta), y_{\text{leg}})$
  for Each component j of $\boldsymbol{x}^{(i-1)}$ **do**
    $\delta_j = \alpha \cdot \text{sign}(g_j^{(i)})$
    $x_j^{(i)} = x_j^{(i-1)} + \delta_j$
    if $x_j^{(i)} > x_j^{(0)} + \gamma$ **then**
      $x_j^{(i)} = \pi_C(x_j^{(i)}) = 2(x_j^{(0)} + \gamma) - x_j^{(i)}$
    end if
    if $x_j^{(i)} < x_j^{(0)} - \gamma$ **then**
      $x_j^{(i)} = \pi_C(x_j^{(i)}) = 2(x_j^{(0)} - \gamma) - x_j^{(i)}$
    end if
  end for
end for
return $\boldsymbol{x}^* = \frac{\boldsymbol{x}^{(T)}}{||\boldsymbol{x}^{(T)}||}$

Momentum iterative method

PGD performs FGSM iteratively with a much smaller step size to deal with rapidly changing gradients, but it can easily drop into local extremums and exhibit poor performance. The momentum iterative method (MIM) introduces momentum into the iterative FGSM to avoid being trapped by local extremums. Concretely, with l_∞-norm ϵ, iteration number T, the MIM updates the adversarial example with the following rule [11]:

$$a_0 = 0, \quad a_t = \mu \cdot a_{t-1} + \frac{\nabla_x L(f(\boldsymbol{x}^{(t-1)};\theta), y_{\text{leg}})}{||\nabla_x L(f(\boldsymbol{x}^{(t-1)};\theta), y_{\text{leg}})||}, \tag{17}$$

$$\boldsymbol{x}^{(t)} = \boldsymbol{x}^{(t-1)} + \frac{\epsilon}{T} \cdot \text{sign}(a_t), \tag{18}$$

where μ is a decay factor and a performs as the accelerated velocity which contains the information of past gradient descent direction. The pseudo-code for MIM is shown in Algorithm 2.

Algorithm 2 Momentum Iterative Method

Input The classifier $f(\bullet;\theta)$, loss function L, legitimate density matrix $(\boldsymbol{x}_{\text{leg}}, y)$.
Input The l_∞-norm restriction ϵ, iteration number T, decay factor μ.
Output An adversarial example $\boldsymbol{x}^*$.

$\boldsymbol{x}^{(0)} = \boldsymbol{x}_{\text{leg}}$
$\alpha = \frac{\epsilon}{T}$
$a_0 = 0$
for $i = 1, \ldots, T$ **do**
  $a_i = \mu \cdot a_{i-1} + \frac{\nabla_x L(f(\boldsymbol{x}^{(i-1)};\theta), y_{\text{leg}})}{||\nabla_x L(f(\boldsymbol{x}^{(i-1)};\theta), y_{\text{leg}})||}$
  $\boldsymbol{x}^{(t)} = \boldsymbol{x}^{(t-1)} + \alpha \cdot \text{sign}(a_t)$
end for
return $\boldsymbol{x}^* = \frac{\boldsymbol{x}^{(T)}}{||\boldsymbol{x}^{(T)}||}$

Differential evolution algorithm

Differential evolution algorithm (DEA) belongs to the general class of evolutionary algorithms, which is powerful to solve complex multi-modal optimization problems. Concretely, DEA first randomly generates n candidates $\{X\} = X_1, X_2, \ldots, X_n$ as possible solutions. These candidates become parents in the first iteration. In each iteration, DEA generates a new set of candidates called children from the current parents first by [12, 13]:

$$X_i' = X_j + F \cdot (X_k - X_l), \tag{19}$$

where X_j, X_k, X_l are randomly picked from $\{X\}$ and distinct from each other. F is called the mutual factor.

Then, for each component j of X_i', with randomly picked r_j from $U(0,1)$:

$$(X_i')_j = (X_i)_j, \quad \text{if } r_j > P, \tag{20}$$

where P is called the crossover probability. If the children X_i' has better performance than the parent X_i, then DEA will replace X_i with X_i'. This procedure will repeat for several iterations until the candidates are almost converged.

In our scenario, we need to discretely change m density matrices on the $10 \times 10 \times 10$ grid and keep others unchanged. Therefore, the candidates are in the form of

$$\begin{aligned} X &= (\mathbf{pos}_1, \mathbf{idx}_1) \times (\mathbf{pos}_2, \mathbf{idx}_2) \times \cdots \times (\mathbf{pos}_m, \mathbf{idx}_m) \\ &= (\mathbf{pos}, \mathbf{idx})^m \end{aligned}, \tag{21}$$

which changes $\boldsymbol{x}_{\text{leg}}$ by setting $(\boldsymbol{x}_{\text{leg}})_{\mathbf{pos}_i} = \mathbf{idx}_i$. The pseudo-code for DEA is shown in Algorithm 3.

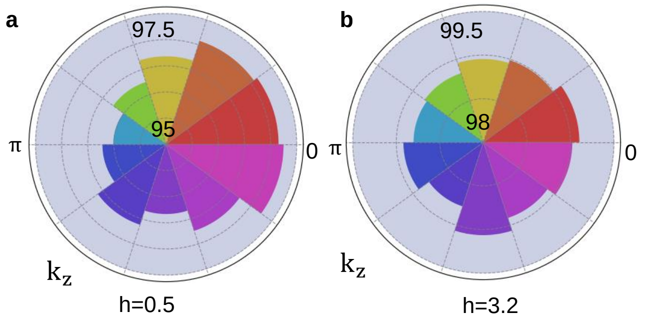

Supplementary Figure 6. **State fidelity $F_{\mathbf{k}}$ between experimentally implemented adversarial examples and legitimate samples.** **a** The fidelity at different $\mathbf{k_z}$ values with $h = 0.5$. The angular direction represents different k_z and the radius direction represents the fidelity. **b** The fidelity at different $\mathbf{k_z}$ values with $h = 3.2$.

We use cleverhans [14] to implement PGD and MIM, and adapt the code in Ref. [15] to implement DEA. We use MIM for legitimate samples with $h = 0.5, 2$ and use PGD for $h = 3.2$ to numerically generate adversarial examples with continuous perturbations on all density matrices. We also use DEA for $h = 3.2$ to numerically generate adversarial examples with only seven discrete density matrices changed. It should be noted that, although all perturbations generated by PGD, MIM, and DEA are restricted to be tiny, they can make the legitimate samples unphysical, namely that they are not normalized to one after adding the perturbation. To avoid this, we renormalize all density matrices in the adversarial examples, but this can make these examples lose the ability to mislead the classifier. The noises in experiment can also deteriorate their performance. To deal with this problem, we repeatedly adjust the parameters and restrictions to numerically generate different adversarial examples until there is one can successfully mislead the classifier after normalization and adding simulated noises. After numerically generating these adversarial examples, we reconstruct their Hamiltonian in the NV center. The fidelity between experimentally realized adversarial examples and legitimate samples at different k_z values are shown in Supplementary Fig. 6. The average fidelity for $h = 0.5$ and $h = 3.2$ are 96.63 % and 98.94 %.

Algorithm 3 Differential Evolution Algorithm

Input The legitimate sample $(\boldsymbol{x}_{\text{leg}}, y)$, trained model $f(\bullet; \theta)$.
Input The iteration number T, the population size n, the number m of pixels to be changed, the mutual factor F, the crossover probability P.
Output An adversarial example $\boldsymbol{x}^*$.

Randomly generate perturbation $X_i = (\mathbf{pos}, \mathbf{idx})^m$ with $\mathbf{pos} \in [0,9]^3$ and $\mathbf{idx} \in [-1,1]^3$ for $i = 1, 2, \ldots, n$.
len $= 6m =$ lengnth of each X_i
Perform X_i on $\boldsymbol{x}_{\text{leg}}$ to obtain $\boldsymbol{x}_i^*$ for $i = 1, 2, \ldots, n$.
for $t = 1, 2, \ldots, T$ **do**
  for $i = 1, 2, \ldots, n$ **do**
    Randomly pick distinct $j, k, l \in [n]/\{i\}$
    $X_i' = X_j + F \cdot (X_k - X_l)$
    for $s = 1, 2, \ldots,$ len **do**
      Randomly pick r from $U(0,1)$
      if $r > P$ **then**
        $(X_i')_s = (X_i)_s$
      end if
    end for
    Perform X_i' on $\boldsymbol{x}_{leg}$ to obtain $\boldsymbol{x}_i^{*\prime}$
    if $L(f(\boldsymbol{x}_i^{*\prime}; \theta), y_{\text{leg}}) > L(f(\boldsymbol{x}_i^*; \theta), y_{\text{leg}})$ **then**
      $X_i = X_i'$
      $x_i^* = x_i^{*\prime}$
    end if
  end for
end for
Find the x_q^* that has the largest $L(f(\boldsymbol{x}_i^{*\prime}; \theta), y_{\text{leg}})$ among $\{x_1^*, x_2^*, \ldots, x_n^*\}$
return $\boldsymbol{x}^* = \frac{x_q^*}{||x_q^*||}$

SUPPLEMENTARY NOTE 7: 3D HOPF FIBRATION REPRESENTATION

In the main text, we present a one layer's cross section of spin textures. The topological properties of the Hopf insulator are fully captured by the 3D spin texture. The 3D spin textures of the realized legitimate and adversarial examples are shown in Supplementary Fig. 7. This result shows that the spin textures of topological nontrivial phases ($h = 0.5$ for $\chi = -2$ and $h = 2$ for $\chi = 1$) are twisted in a nontrival way, while the topological trivial one ($h = 3.2$ for $\chi = 0$) is untwisted. The nonzero integer-valued topological invariant guarantees the spin texture cannot be untwisted by continuous deformations. Comparing with corresponding adversarial

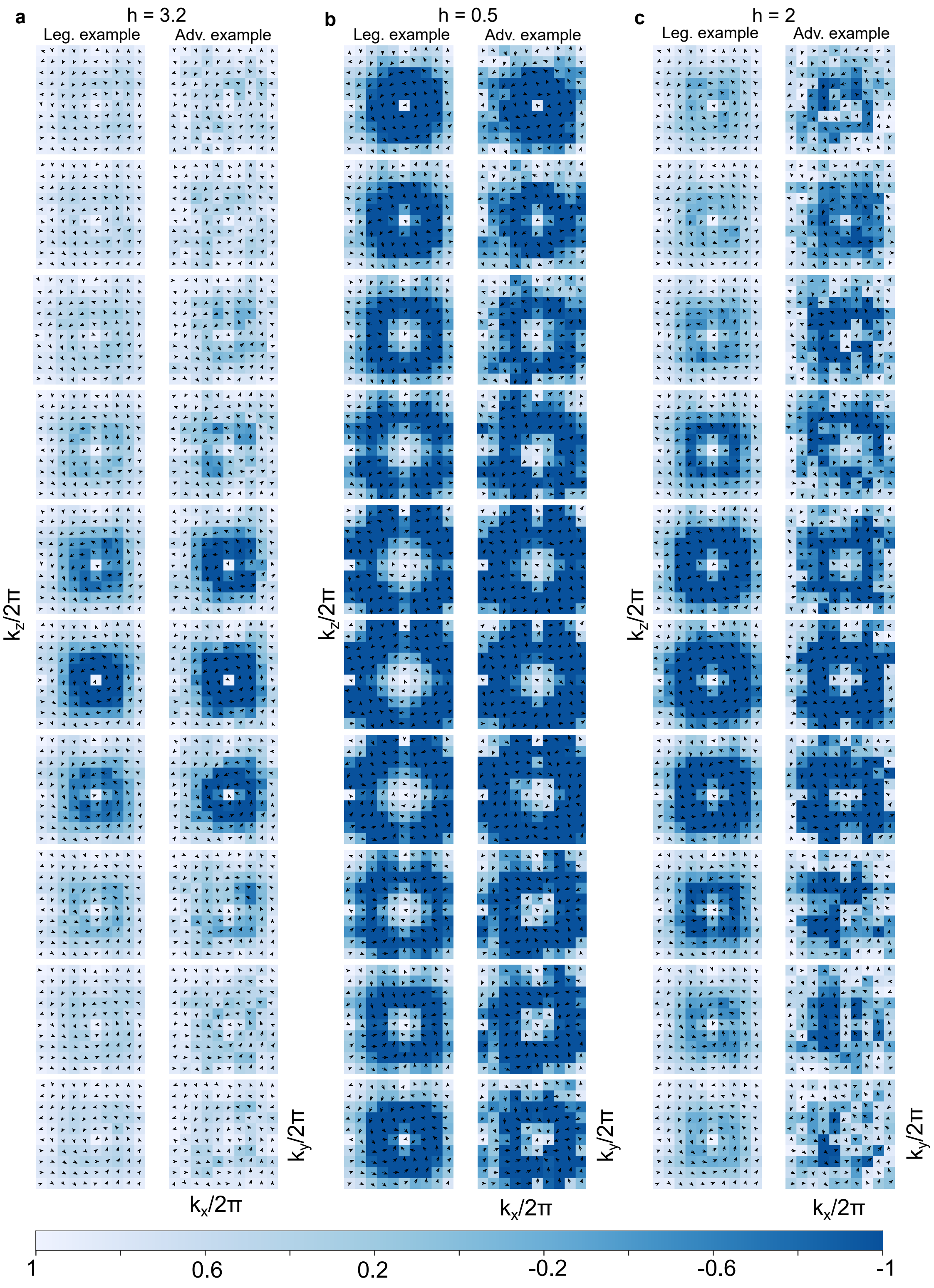

Supplementary Figure 7. Measured spin textures of experimental legitimate samples and adversarial examples obtained by continuous attacks. Each column contains all layers of measured spin textures for $k_z = 0, 0.1, \ldots, 0.9 \times 2\pi$. The arrow at each point shows the projection of the Bloch vector into the x-y plane and the color represents the magnitude of the z component of the Bloch vector. **a** Topologically trivial phase with $h = 3.2$, corresponding to $\chi = 0$. **b** Topological nontrivial phase with $h = 0.5$, corresponding to $\chi = -2$. **c** Topological nontrivial phase with $h = 2$, corresponding to $\chi = 1$.

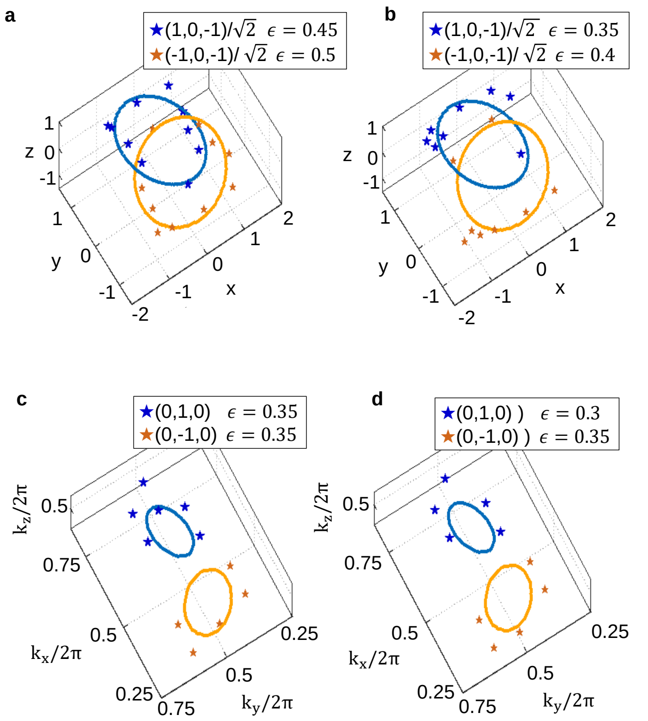

Supplementary Figure 8. **The 3D preimage contours that show topological properties of the Hopf insulator. a** Topological link, obtained from two spin states with Bloch sphere representation $\mathbf{S} = (1, 0, -1)/\sqrt{2}$ (blue) and $(-1, 0, -1)/\sqrt{2}$ (orange), for the legitimate sample with $h = 2$ in the stereographic coordinates. Solid lines are curves from theoretically calculated directions $\mathbf{S_{th}}$ and the stars are experimentally measured spin orientations. The deviation $|\mathbf{S_{exp}} - \mathbf{S_{th}}| \leq 0.45$ (0.5) for the blue (orange) curve. **b** Topological link of adversarial examples with $h = 2$ with deviation $|\mathbf{S_{exp}} - \mathbf{S_{th}}| \leq 0.35$ (0.4) for blue (orange) curves. **c** Unlinked loops of legitimate sample with $h = 3.2$. Two spin orientations are $\mathbf{S} = (0, 1, 0)$ (blue) and $\mathbf{S} = (0, -1, 0)$ (orange). The deviation $|\mathbf{S_{exp}} - \mathbf{S_{th}}| \leq 0.35$ for the blue and orange curve. **d** Unlinked loops of adversarial example of $h = 3.2$. The deviation $|\mathbf{S_{exp}} - \mathbf{S_{th}}| \leq 0.3$ (0.35) for the blue (orange) curve.

examples, it is evident that although adversarial perturbations can modify the local details of the spin textures, they do not change its global twisted features and thus the corresponding topological index remains unaltered.

From the Eq. (1) in the main text, we can derive the ground state on the Bloch sphere $\mathbb{S}^2$ with any given momentum point $\mathbf{k}$ in $\mathbb{T}^3$. Consequently, by this equation, one can obtain the preimage contours in $\mathbb{T}^3$ of any spin orientation $\mathbb{S}^2$. To visualize the link and knot for topological nontrivial phases without gluing the boundary, we map the preimage contours in $\mathbb{T}^3$ into $\mathbb{R}^3$. Concretely, the mapping can be decomposited into two parts [6]:

$$\mathbb{T}^3 \xrightarrow{g} \mathbb{S}^3 \xrightarrow{s} \mathbb{R}^3. \qquad (22)$$

The mapping g first maps the 3D torus $\mathbb{T}^3$ to the stereographic coordinates $\mathbb{S}^3$ by:

$$\eta_\uparrow(\mathbf{k}) = \sin k_x - i \sin k_y,$$
$$\eta_\downarrow(\mathbf{k}) = \sin k_z - i(\cos k_x + \cos k_y + \cos k_z + h). \qquad (23)$$

where $(\eta_1, \eta_2, \eta_3, \eta_4) = (\mathrm{Re}[\eta_\uparrow],\ \mathrm{Im}[\eta_\uparrow],\ \mathrm{Re}[\eta_\downarrow],\ \mathrm{Im}[\eta_\downarrow])$ are points on $\mathbb{S}^3$ (up to a trivial normalization). For the purpose of easy visualization, the links are further transformed from the stereographic coordinates of $\mathbb{S}^3$ to $\mathbb{R}^3$ by mapping s:

$$(x, y, z) = \frac{1}{1 + \eta_4}(\eta_1, \eta_2, \eta_3). \qquad (24)$$

In Fig. 4 in the main text, we show the 3D preimage contours of topological nontrivial phase $\chi = -2$, which present topological links between two orientations, keep twisted together after adding the perturbations to legitimate samples. The two orientations of spins in Bloch sphere chosen in the figure are $\mathbf{S_{th}} = (1, 0, -1)/\sqrt{2}$ and $(-1, 0, -1)/\sqrt{2}$. To obtain the preimage contours on a discrete momentum data grid, we need to select all preimage points with a prescribed tolerance threshold. This can be achieved by defining an ϵ-neighborhood of the desired spin orientation $\mathbf{S}_{\mathrm{th}}$ [16]:

$$N_\epsilon(\mathbf{S}_{\mathrm{th}}) = \{\mathbf{S}(\mathbf{k}) : |\mathbf{S}(\mathbf{k}) - \mathbf{S}_{\mathrm{th}}| \leq \epsilon\}. \qquad (25)$$

The choice of ϵ satisfies condition containing sufficient data points and displaying a clear loop structure simultaneously. We use the same method and obtain preimages in momentum space $\mathbb{T}^3$ for topological trivial phase $\chi = 0$ in Supplementary Fig. 8**a - b** and topological nontrivial phase $\chi = 1$ in Supplementary Fig. 8**c - d**. From this figure one can clearly figure out that adversarial perturbations do not change the topological links: for topological nontrivial phases the preimage countours are linked whereas for the trivial ones they are unlinked.

SUPPLEMENTARY NOTE 8: ADVERSARIAL TRAINING

As mentioned in the main text, in order to enhance the robustness of the phase classifier to adversarial perturbations, we have tried a defense strategy that is a variation of adversarial training. Adversarial training [10], which is the simplest and most straightforward one among various defense strategies, is to train the classifier by legitimate and adversarial data alternatively. To increase the classifier's robustness to both carefully designed and experimental noises, we modify the adversarial training algorithm and make it also take the randomly dropped data into account. The algorithm we use is shown in Algorithm 4.

After the adversarial training, the classifier becomes immune to both the adversarial attack M used in adversarial training and the experimental noises when a large

portion of data are dropped. As shown in Supplementary Fig. 9**a**, we test the experimental implementation for legitimate sample with $h = 3.2$ under the same condition as the case in the main text. We find that after adversarial training, the classifier can correctly classify the sample with more than 90% percent of data are dropped, and the ratio of adversarial perturbations in the experimental noises increases much slower. This result may arise from two reasons: i) adding the randomly dropped data in training dataset forces the classifier to learn the relation between different density matrices in the $10 \times 10 \times 10$ grid. ii) adding the adversarial examples with small perturbations in training dataset forces the classifier to obtain more flatten weights and make similar predictions near each data point.

Although this strategy can improve the classifier's robustness to both kinds of adversarial perturbations in most cases, it decreases the classifier's performance near the phase transition points: as shown in Supplementary Fig. 9**b**, after adversarial training, the classifier's confidence cliff around the phase transition point becomes flatter. This result is naturally originated from the reason ii) mentioned above, which indicates that the classifier is forced to perform similarly near the transition point. Actually, the phenomenon that there is a competition between adversarial robustness and generalization accuracy, is known as the "No free lunch" theorem for adversarial robustness: any classifier can be misled with high confidence once the perturbations are slightly greater than the natural noises if class labels and the data distribution satisfies certain conditions.

Algorithm 4 Adversarial Training

Input The training dataset $\mathcal{D} = (\boldsymbol{x}, y)$, the loss function L, the training epochs T, the batch size B, the gradient optimization methods G.
Input The method M for generating adversarial examples.
Output An adversarial trained classifier f.

Initialize the classifier $f = f(\bullet, \theta_1)$
$n = \lfloor \frac{|\mathcal{D}|}{B} \rfloor$
for $i = 1, \ldots, T$ **do**
  for $j = 1, \ldots, n$ **do**
    Choose next B samples from $\mathcal{D}$:$\{\boldsymbol{x}^{(B)}, y^{(B)}\}$
    $l_{\text{leg}} = L(f(\boldsymbol{x}^{(B)}, \theta_i), y^{(B)})$
    $\boldsymbol{x}^{(B)}_{\text{adv}} = M(f(\bullet, \theta_i), \boldsymbol{x}^{(B)})$
    $l_{\text{adv}} = L(f(\boldsymbol{x}^{(B)}_{\text{adv}}, \theta_i), y^{(B)})$
    Initialize $\boldsymbol{x}^{(B)}_{\text{drop}} = \boldsymbol{x}^{(B)}$
    Dropping number $N = \lfloor U(0, 10 \times 10 \times 10) \rfloor$
    for $k = 1, \ldots, N$ **do**
      Dropping index idx $= \lfloor U(0, 10 \times 10 \times 10) \rfloor$
      $\boldsymbol{x}^{(B)}_{\text{drop}}(\text{idx}) = 0$
    end for
    $l_{\text{drop}} = L(f(\boldsymbol{x}^{(B)}_{\text{drop}}, \theta_i), y^{(B)})$
    $l^{(B)} = 0.5 l_{\text{leg}} + 0.25 l_{\text{adv}} + 0.25 l_{\text{drop}}$
    update $\theta_{i+1} = \theta_i - G(f(\bullet, \theta_i), l^{(B)})$
  end for
return $f = f(\bullet, \theta_{T+1})$

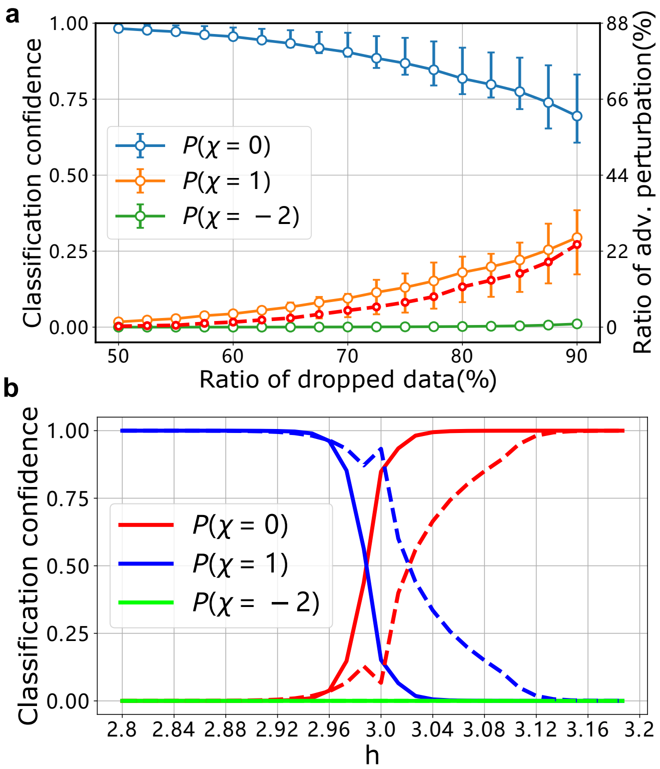

Supplementary Figure 9. **The performance for the adversarial training. a** After adversarial training, the classifier can correctly classify the clean data of topological trivial phase ($h = 3.2, \chi = 0$) with more than 90% of the data dropped; and the ratio of adversarial (Adv.) perturbations also increases much slower compared with the case Fig. 1**c** in the main text. The error bars are obtained from 100 random data dropping trials. **b** The classification confidence cliff near the phase transition point $h = 3$. The solid lines indicate the classifier's confidence before adversarial training. The dash lines indicate the classifier's confidence after adversarial training. After adversarial training, the confidence cliff near the transition point becomes flatter, which would affect the accurate identification of the phase transition points.

SUPPLEMENTARY REFERENCES

[1] Epstein, R. J., Mendoza, F. M., Kato, Y. K. & Awschalom, D. D. Anisotropic interactions of a single spin and dark-spin spectroscopy in diamond. *Nat. Phys.* **1**, 94–98 (2005).

[2] Lian, W.-Q. *et al.* Machine learning topological phases with a solid-state quantum simulator. *Phys. Rev. Lett.* **122**, 210503 (2019).

[3] James, D. F., Kwiat, P. G., Munro, W. J. & White, A. G. Measurement of qubits. *Phys. Rev. A* **64**, 052312 (2001).

[4] Fukui, T., Hatsugai, Y. & Suzuki, H. Chern numbers in discretized brillouin zone: Efficient method of computing

(spin) hall conductances. *J. Phys. Soc. Jpn* **74**, 1674–1677 (2005).

[5] Deng, D.-L., Wang, S.-T. & Duan, L.-M. Direct probe of topological order for cold atoms. *Phys. Rev. A* **90**, 041601 (2014).

[6] Yuan, X.-X. *et al.* Observation of topological links associated with hopf insulators in a solid-state quantum simulator. *Chin. Phys. Lett.* **34**, 060302 (2017).

[7] Carrasquilla, J. & Melko, R. G. Machine learning phases of matter. *Nat. Phys.* **13**, 431–434 (2017).

[8] van Nieuwenburg, E. P. L., Liu, Y.-H. & Huber, S. D. Learning phase transitions by confusion. *Nat. Phys.* **13**, 435–439 (2017).

[9] Ch'ng, K., Carrasquilla, J., Melko, R. G. & Khatami, E. Machine learning phases of strongly correlated fermions. *Phys. Rev. X* **7**, 031038 (2017).

[10] Madry, A., Makelov, A., Schmidt, L., Tsipras, D. & Vladu, A. Towards deep learning models resistant to adversarial attacks. In *6th International Conference on Learning Representations* (2018).

[11] Dong, Y.-P. *et al.* Boosting adversarial attacks with momentum. In *Proceedings of the IEEE Conference on Computer Vision and Pattern Recognition*, 9185–9193 (2018).

[12] Storn, R. & Price, K. Differential evolution – a simple and efficient heuristic for global optimization over continuous spaces. *Journal of Global Optimization* **11**, 341–359 (1997).

[13] Das, S. & Suganthan, P. N. Differential evolution: A survey of the state-of-the-art. *IEEE Transactions on Evolutionary Computation* **15**, 4–31 (2011).

[14] Papernot, N. *et al.* Technical report on the cleverhans v2. 1.0 adversarial examples library. Preprint at https://arxiv.org/abs/1610.00768 (2018).

[15] Su, J., Vargas, D. V. & Sakurai, K. One pixel attack for fooling deep neural networks. *IEEE Transactions on Evolutionary Computation* **23**, 828–841 (2019).

[16] Deng, D.-L., Wang, S.-T., Sun, K. & Duan, L.-M. Probe knots and hopf insulators with ultracold atoms. *Chin. Phys. Lett.* **35**, 013701 (2018).
